# Erdheim-Chester Disease: a comprehensive review of the literature

**DOI:** 10.1186/1750-1172-8-137

**Published:** 2013-09-08

**Authors:** Roei D Mazor, Mirra Manevich-Mazor, Yehuda Shoenfeld

**Affiliations:** 1 The Zabludowicz Center for Autoimmune Diseases, Sheba Medical Center, Tel Hashomer, Israel; 2 Sackler Faculty of Medicine, Tel Aviv University, Tel Aviv, Israel; 3 The Laura Schwarz-Kipp Chair for Research of Autoimmune Diseases, Tel-Aviv University, Tel-Aviv, Israel

**Keywords:** Erdheim Chester disease, Interferon alpha, Interleukin-1, BRAF

## Abstract

Erdheim-Chester Disease (ECD) is a rare form of non Langerhans' cell histiocytosis. Individuals affected by this disease are typically adults between their 5th and 7th decades of life. Males and females are almost equally affected. The multi systemic form of ECD is associated with significant morbidity, which may arise due to histiocytic infiltration of critical organ systems. Among the more common sites of involvement are the skeleton, central nervous system, cardiovascular system, lungs, kidneys (retroperitoneum) and skin. The most common presenting symptom of ECD is bone pain. The etiology of ECD is unknown yet thought to be associated with an intense TH1 immune response. It may also be associated with the V600E BRAF mutation, as described in as many as half of the patients in recent studies. Bilateral symmetric increased tracer uptake on ^99m^Tc bone scintigraphy affecting the periarticular regions of the long bones is highly suggestive of ECD. However, definite diagnosis of ECD is established only once CD68(+), CD1a(−) histiocytes are identified within a biopsy specimen. At present, this obscure ailment embodies numerous challenges to medical science. Given its rarity, it is diagnostically elusive and requires a high level of clinical suspicion. Therapeutically, it is of limited alternatives. Currently, interferon-α is the most extensively studied agent in the treatment of ECD and serves as the first line of treatment. Treatment with other agents is based on anecdotal case reports and on the basis of biological rationale. Nevertheless, cladribine (2CDA), anakinra and vemurafenib are currently advocated as promising second line treatments for patients whose response to interferon-α is unsatisfactory. Overall, the 5 year survival of ECD is 68%. Herein, the authors mustered and brought about a panoramic consolidation of all the relevant facts regarding ECD. This work highlights the different clinical, radiological and pathological manifestations associated with ECD, the differential diagnoses, the various treatment options and the acknowledged science explaining the disease.

## Introduction

### History, Classification, Epidemiology, Etiology and Prognosis

Erdheim-Chester Disease (ECD) is a rare form of non Langerhans' cell histiocytosis originally described as "Lipid Granulomatosis" in 1930 by Jakob Erdheim and William Chester. As of present time, only several hundred cases had been documented in the medical literature [1], the majority of which were described in the past ten years [2]. The chronicles of the disease encompass a variety of pathophysiological processes and diverse clinical manifestations originating from the infiltration of lipid-laden histiocytes with foamy or eosinophilic cytoplasm to bones and various organs. The heterogeneous manifestations of ECD vary amongst different individuals. This results in a presentation that may vary from an indolent focal disease to a life threatening organ failure [3]. Although ECD primarily affects adults between their 5th and 7th decades of life [4] patients have been diagnosed between the ages of 7 to 84 years [5] and pediatric cases have been documented in the medical literature [6-9]. A slight male predominance was noted amongst patients [10] and some studies suggest that male patients are diagnosed at a more advanced age than female patients [11]. The etiology of the disease is unknown [12] and considered to be non-genetic and not associated with an infectious agent [11,13]. The scarcity of patients serves as an obstacle in medical science's endeavor to better understand this condition, rendering the formulation of controlled randomized trials - impossible. Researchers face a constant shortage of relevant biological samples. Despite recent advancements, the pathogenesis of this disease is still poorly understood. The broad and complex manifestations of ECD, in conjunction with its rarity and with only a handful of centers of referral in the world may inevitably lead to misdiagnosis. Physicians mainly rely on retrospective data describing the clinical course of the disease as published in former case reports and literature reviews. Consequently, current treatments are based on an anecdotal evidence base and the overall prognosis is grim. Arnaud et al. [1] report of the 1-year and 5-year survival rates to be 96% and 68% respectively. Many academic debates had risen in the past concerning the proper classification of ECD. Initially, it was thought to be a variant of Langerhans' Cell Histiocytosis (LCH) [14,15] and as time went by, ECD developed a unique identity as a singular disease entity in the medical literature, baring specific diagnostic criteria. When referring to histiocytic disorders, it is comfortable to classify them into Langerhans' Cell Histiocytoses (or "X Type" Histiocytoses) and Non-Langerhans Cell Histiocytosis (or "Non X Type" HIstiocytoses). While the former includes diseases such as Hand-Schuller-Christian disease, Letterer-Siwe disease, and eosinophilic granuloma [5], the latter includes diseases such as Erdheim-Chester disease (ECD) [5] and Juvenile xanthogranuloma (JXA) [16]. In respect to the above, one cannot ignore the common properties of histiocytic disorders in general and of LCH and ECD in particular. Present knowledge of the pathogenesis and etiology of ECD is very little, much is unknown and many questions remain unanswered. Interesting tangency points between LCH and ECD exist [17,18] both diagnostically and therapeutically, suggesting a common denominator of both conditions. This fact is even more substantiated in the face of patients diagnosed with both diseases at the same time [19-23].

### Diagnostic criteria

**Figure 1:**
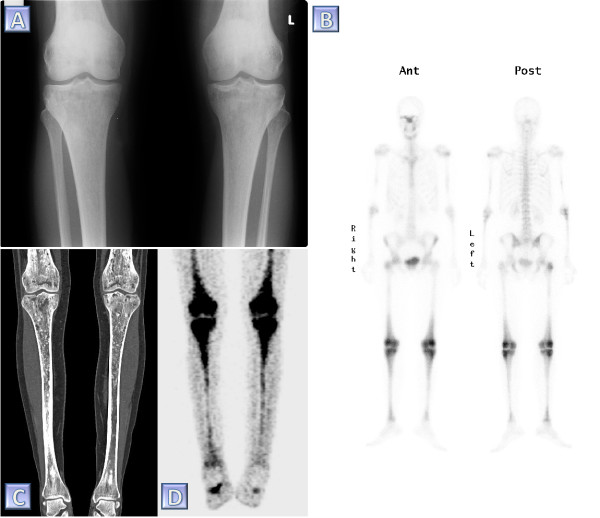
**Diagnostic imaging in ECD. Various modalities in the skeletal assessment of a single ECD patient. (A)** A plain radiograph of the knees demonstrating bilateral sclerotic changes in the femoral and tibial bones. **(B) **^99m^Tc bone scintigraph taken prior to the diagnosis of ECD. Note the abnormally increased tracer uptake especially involving the periarticular region of the femurs and the tibiae. **(C)** Coronal reconstruction of a computed tomography study of the femurs and tibiae. Note the diffuse, irregular intra-medullary lytic-sclerotic pattern as well as the marked cortical thickening of the tibiae. **(D)** Coronal reconstruction of a positron emission tomography taken for the purpose of follow up 4.5 years pursuant the diagnosis of ECD. This study shows bilateral symmetric abnormally increased intra-medullary uptake of fluorodeoxyglucose in the femurs and tibiae.

Diagnosis of ECD relies on established radiological and histological criteria. The major criterion is the distinct histological pattern that characterizes the condition [24] which is usually obtained after the radiological findings, which in turn are prompted by the patient’s symptoms. Thus, typical histological findings are sufficient to confirm ECD. Considering the rarity of this condition, the necessity for a pathognomonic signature of the disease seems abundantly clear. The most common presenting symptom of ECD is bone pain. When submitting the absolute majority of ECD patients to imaging studies, characteristic radiographic changes in the long bones appear (Figure 1). These changes, namely, bilateral cortical sclerosis involving the diametaphyseal regions, are considered virtually pathognomonic [25]. Two relevant types of imaging studies are radiographs and ^99m^Tc bone scintigraphs. In ECD, bilateral symmetric osteosclerotic lesions are typically observed on radiographs, while abnormally strong labeling of the distal ends of the long bones are observed on ^99m^Tc bone scintigraphs. Detecting either of those comprises the radiological diagnostic criterion. The histological diagnostic criterion is met providing that typical ECD histiocytes are found in the examined lesion. These histiocytes are non langerhans' foamy histiocytes, which lack Birbeck granules, nested within a polymorphic granuloma, fibrosis or xanthogranulomatosis. Immunohistochemical staining is positive for CD68 and negative for CD1a [26]. Further elaboration on the microscopic, ultrastructure and immunohistochemical properties of the typical histological findings appear in Table 1.

**Table 1:** Diagnostic criteria of ECD

Radiology	Radiography	Bilateral symmetric diametaphyseal osteosclerosis of long bones
	^99m^Tc Bone Scintigraphy	Symmetric and abnormally strong 99mTc labeling of the distal ends of the long bones
Histology	Microscopic Environment	Non Langerhans histiocytes with foamy or eosinophillic cytoplasm, polymorphic granulomae and fibrosis, xanthogranulomatosis, proliferating fibroblasts, lymphocytic aggregates, Touton giant cells
	**Histiocyte Immunostaining**	CD68(+), CD1a(−), S-100(negative/low)^*^
	Histiocyte Ultrastructure	Lack of Birbeck granules

(*) Pathological confirmation of CD68(+), CD1a(−) histiocytes is both sufficient and mandatory for the diagnosis of ECD.

### General symptoms

A variety of general symptoms may accompany ECD. These are relatively unspecific and do not appear globally in all patients. However, when apparent, they may serve as means for evaluating the patient’s well being and provide gross assessment of the patient’s response to treatment. Amongst them are fever, weakness, weight loss [5] and night sweats [27]. Fatigue may be associated with a microcytic anemia, which occasionally accompanies ECD [28]. Additionally, pediatric cases of ECD may present with a failure to thrive [9].

### Skeletal involvement

Involvement of the skeleton occurs in up to 96% of ECD patients. Bone pain, however, occurs in only 50% of the cases [2]. The most frequently affected bones are the femur, tibia and fibula and less frequently the ulna, radius and humerus. Bone pain usually manifests around the knees and ankles. Osteosclerosis occurs bilaterally and symmetrically in the diametaphyseal regions of the long bones. The axial skeleton and epiphyseal regions are usually spared [29]. However, Dion et al. [30] reported partial epiphyseal involvement and evidence of periostitis upon a thorough radiological survey of 11 ECD patients. While the classical hallmark of the skeletal involvement of ECD is osteosclerosis, occasionally, mixed sclerotic and lytic lesions have been described. It is imperative to recognize that bone lesions found in LCH are rather lytic than sclerotic [31] further complicating the diagnosis in cases which the typical bone changes are a tad less typical, in conjunction with osteolytic lesions. In their retrospective study encompassing 59 cases of ECD in 1996, Veyssier-Belot et al. reported that 5%-8% of the patients in the study also had lytic lesions, either on the flat bones, like the ribs and skull, or on the long bones [5]. On 2002, Oweity et al. reported that 30% of ECD cases exhibit osteolytic lesion involvement [13]. In addition, both typical and atypical ECD lesions may present in atypical foci [32,33]. Both the common and uncommon radiological findings of ECD related osseous involvement should be considered in the differential diagnosis alongside other medical conditions - on a case by case basis. These include osteomyelitis, Paget's disease, lymphoma, sarcoidosis, bony metastases [33] and lipid storage diseases such as Gaucher's disease and Niemann-Pick disease [34]. The skeletal involvement seldom appears alone. The retrospective study of 59 ECD cases discussed above, reported that approximately 50% of ECD patients had extraskeletal manifestations upon diagnosis [5]. Moreover, Arnaud et al. recently reported that 98% of the 53 patients they reviewed had at least one extraskeletal manifestation of the disease [1]. In summary, the diagnosis of ECD is usually made on the basis of bone pain and pathognomonic radiological features. However, it may turn challenging due to the abundance of possible extraskeletal involvement sites. Particularly in cases in which the patient’s chief complaints deviate the physician’s attention from the skeleton towards other foci of the disease.

### Central nervous system involvement

Progression of ECD to the CNS and adjacent structures, such as the meninges, facial bones, orbits and intracranial vasculature can manifest in a wide range of symptoms. The location, size and nature of the lesion at hand determine whether the patient will be completely asymptomatic, suffer from various neurological deficits, severe disability or succumb to his disease. CNS involvement appears in approximately 51% of ECD patients and accounts for 29% of all deaths, as reported by Arnaud et al. [1]. In another retrospective analysis of 33 patients with confirmed ECD, performed by Drier et al., 45% of the patients had symptoms related to CNS and/or orbital manifestations at presentation. These manifestations, by order of frequency, were diabetes insipidus, exophthalmos, cerebellar ataxia, panhypopituitarism and papilledema [10]. The manifestations mentioned above correlate with diverse radiological and pathological findings: involvement the hypothalamic-pituitary axis where nodular or micronodular masses of the infundibular stalk may be present, retro-orbital masses, involvement of the dentate area of the cerebellum and meningeal lesions of the dura. Other involvements reported in the literature include thickening of the bones of the face and skull, intracranial peri-arterial infiltration, intraluminal involvement of the superior sagittal sinus [10], involvement of the choroid plexus [35] and masses involving the cerebral hemispheres. However, clinically evident deficits in cerebral function are less common [13]. Any type of lesion rarely appears alone; about 66% of the patients examined by Drier et al. had simultaneous involvement of at least two anatomical sites. An association was noted between facial bone osteosclerosis and orbital or meningeal masses. This could serve to direct the physician towards the diagnosis of ECD in the appropriate settings. Lumbar puncture is not recommended since ECD histiocytes rarely appear in the cerebrospinal fluid [36]. The differential diagnosis of ECD related intracranial lesions is wide: Intracerebral histiocytic lesions may mimic a neoplasm of glial origin [37]. Lesions of the cerebellum and brainstem may appear similar to demyelinating diseases such as multiple sclerosis [38]. ECD related suprasellar lesions should be considered in the differential diagnosis alongside Langerhans cell histiocytosis or other adenomatous, granulomatous or inflammatory processes of this region [3]. ECD related retro-orbital involvement may resemble the retro-orbital involvement seen in Wegener’s granulomatosis [10]. In the appropriate clinical settings, Graves disease, Langerhans cell histiocytosis, lymphoma, sarcoidosis, and Sjogren’s disease should also be considered in this context [10,39]. An ECD meningeal lesion may be mistaken for a meningioma [10,40,41] and ECD sinonasal lesions may mimic rhinoscleroma [42].

Central diabetes insipidus is the most common of all CNS manifestations in ECD. It should be noted that histiocytosis associated diabetes insipidus appears early in the natural history of these diseases in general and in ECD in particular [43]. In some cases, even a decade or longer passes before establishing a diagnosis [13] and thus, a high level of suspicion is required. Hypopituitarism and hyperprolactinemia may follow ECD associated dysfunction of the hypothalamus-pituitary axis as well. It is hypothesized that hyperprolactinemia results from mechanical disruption of the hypothalamic dopaminergic prolactin inhibiting pathway, possibly due to compromise of the hypothalamo-pituitary portal system. Hypopituitarism may manifest in different ways, including disruption of thyroid function and gonadotropin balance. In all these cases, MRI may demonstrate a mass in the pituitary stalk or an absence of signal of the posterior pituitary on T1 weighted images. Upon a histological examination of the anterior pituitary, prolactin cell hyperplasia may appear in the adenohypophysis of patients with disseminated ECD [3].

ECD related retro-orbital lesions may present bilaterally or unilaterally, extraconal or intraconal. Most cases however are bilateral and intraconal [10]. These lesions may manifest clinically as exophthalmos. Osteosclerosis of the facial bones commonly presents among ECD patients with orbital lesions or with meningeal lesions. Orbital lesions typically lack signal intensity on both T1 and T2 weighted MR images. Mass effect of the retro-orbital lesions might result in thickening and tortuosity of the optic nerves. The lacrimal glands and orbital muscles, as well as the retro-orbital adipose tissue may be involved with the lesions. Also, periorbital cutaneous xanthomas of yellowish color may appear [39,44].

ECD associated cerebellar syndrome can develop over several years [45]. Additionally, unlike most intracranial lesions, cerebellar lesions appear to have no mass effect. Histological examination of these lesions reveals extensive loss of myelin sheath with gliosis and marked sparing of axons [37,46]. A variety of symptoms correlate with brainstem and cerebellar involvement of ECD. Among them are ataxia, pyramidal syndrome [10,28], cerebellar dysarthria, multidirectional nystagmus, cerebellar dysmetria, hypermetric saccades, negative suppression of the vestibulo-ocular reflex [47] and dysdiadochokinesis [45]. On MRI, these lesions typically appear to be characterized by high signal intensity on T2 weighted images, low signal intensity on T1 weighted images and no enhancement pursuant administration of contrast material [10,23,47].

Drier et al. reported the existence of intracranial peri-arterial lesions in approximately 10% of the ECD patients they had reviewed. As noted before, the lesion-symptom relations are hardly anticipatable and a presence of a lesion does not necessarily translate into a symptom. However, one cannot ignore the possible ischemia and\or thromboembolic complications which may theoretically accompany such a lesion. Therefore, it is recommended to closely monitor such lesions [10].

### Cardiac involvement

Much like ECD involvement of the CNS, cardiovascular involvement of the disease confers a reduced response to treatment and an overall poor prognosis [48]. Approximately 75% of ECD patients suffer from cardiovascular manifestations and about 60% of them will perish due to cardiac complications [49]. The cardiovascular patterns evident in ECD vary depending on the location and size of the lesions. These lesions account for various clinical consequences - congestive heart failure, myocardial infarction, thromboembolism, cardiac remodeling, valvular dysfunction, ischemia, peripheral edema and others. Pericardial infiltration is the most frequent cardiac manifestation of ECD [50]. Both aortic and pericardial involvements of ECD are seen well on computed tomography and echocardiography. Pericardial involvement in ECD takes several forms and symptoms are correlated with the type, extent and severity of the discussed involvement. Upon inspection of the imaging studies, a thickened pericardium may appear with or without an envelope of fibrosis. Pericarditis can be identified by transthoracic echocardiography. A buildup of percardial effusion may lead to cardiac tamponade. This may be relieved by pericardiocentesis which bares diagnostic and therapeutic purposes. Aggressive interventions may include placement of a pericardial window, pericardectomy and placement of a pericardial-abdominal shunt. In one case a notable pericardial effusion in ECD has been reported to mimic a disseminated malignancy [51]. A cytological examination of the pericardial effusion may reveal mesothelial cells, histiocytes and inflammatory cells. A histological examination of a pericardial sample may reveal infiltration of foamy histiocytes [52]. Myocardial involvement follows the pericardial involvement in frequency and presents mainly with myocardial hypertrophy and thickening. This can be seen on echocardiography. Among the different sites of myocardial involvements are the ventricular walls, atrial walls, coronary sulcus [52] and interatrial septum [53]. Involvement of the right atrium with pseudo-tumoral infiltration and involvement of the auriculoventricular sulcus were found at high frequencies [54]. A case series reviewed by Haroche et al. included six patients who presented with a right atrial tumor. In some of the cases reported in the literature, the right atrial tumor was found to traverse the entire atrial wall from the endocardium to the epicardium, thus, disrupting normal cardiac function. It appears that this particular process begins in the subepicardial fatty tissue and progresses into the myocardium. The differential diagnosis of an ECD associated cardiac tumor includes mainly a cardiac myxoma [55]. In an article discussing the myocardial involvement of ECD, Loeffler et al. stress that atrial myocardial involvement in ECD should be recognized as a significant contributor to the patient's morbidity [56]. Infiltration of the myocardium by histiocytes and multi-nucleated giant cells are probable biopsy findings in these cases. The valvular involvement of ECD has been known to cause mainly aortic and mitral regurgitation, preceding the cardiac and hemodynamic consequences and natural history of such insufficiencies [49,57]. Both an MRI and a gated CT scan of the heart are valuable tools in assessing cardiovascular involvement in ECD [54]. Among the ECD related ECG abnormalities observed were short PR segments, sinoauricular blocks, sinus bradycardia, MI compatible Q wave abnormalities with no past MI, ST-T abnormalities and a slight ST elevation [54].

### Coronary & great vessels involvement

Perivascular infiltration in general and periaortic fibrosis specifically, are among the most frequent cardiovascular lesions in ECD and often worsen the prognosis [10]. Periaortic fibrosis appears as a "coated aorta" on CT scans. The degree of periaortic fibrosis alters between patients and anatomical locations. The lesion described can be symmetric or non symmetric, circumferential or non circumferential. It can be limited to a specific segment of the aorta or affect the entire vessel. The "coated aorta" phenomenon is the result of a periaortic infiltration by histiocytes, predominantly to the adventitia. However, it was also suggested that in some cases ECD histiocytes infiltrate as deep as the intimal layer of the aorta. This is not common, but further substantiated by an irregular appearance of the intima on CT among certain ECD patients with aortic involvement - a finding that is so far pathognomonic to ECD [52]. The differential diagnosis of a "coated aorta" appearance on CT includes retroperitoneal fibrosis and Takayasu arteritis. Thus, such a CT finding should prompt a bone scan which might be helpful in substantiating a diagnosis of ECD [58]. Perivascular infiltration is not limited only to the aorta, as it has been identified in large vessels adjacent to the aorta, including but not limited to the brachiocephalic trunk, left common carotid artery, left subclavian artery, coronary arteries, pulmonary trunk, celiac trunk, superior mesenteric artery, and renal arteries [52]. It is hypothesized that this occurs due to the diffuse nature of the xantogranulomatous process affecting the vessels. Lesion associated arterial stenosis was reported regarding the abdominal aorta, celiac trunk, superior mesenteric artery and left renal artery. Hence, vascular involvement in ECD may lead to dire clinical consequences, particularly when arterial stenosis is involved. Such consequences may include cerebral ischemia due to carotid involvement, myocardial infarction due to coronary involvement, mesenteric ischemia due to superior mesenteric artery involvement and renovascular hypertension due to ostial stenosis of renal arteries [52]. Renovascular hypertension usually resolves pursuant angioplasty and stenting of the stenotic renal artery. Coronary artery involvement has been occasionally reported in the medical literature. In one such case, a transmural involvement of all three coronary arteries was noted. In these arteries, the lumen was severely stenosed or obliterated by a yellowish white intimal plaque. This plaque was laden with the typical ECD CD68(+) CD1a(−) histiocytes [59]. ECD associated venous involvement is much less common. Corresponding anecdotal cases describe deep vein thrombosis, pulmonary embolism, sagittal sinus thrombosis and obstruction of the superior vena cava [52]. Involvement of the coronary sinus was also noted [52].

### Pulmonary involvement

A definite diagnosis of pulmonary ECD relies on the detection of typical histiocytic infiltrates in the lung. ECD associated pulmonary involvement was reported to occur in 43% of the patients reviewed by Arnaud et al. [1]. ECD is a cause of interstitial lung disease [60]. Thus, pulmonary ECD is considered highly probable when identifying the radiological hallmarks of interstitial lung disease in ECD patients. The differential diagnosis of ECD associated interstitial lung disease includes usual interstitial pneumonia, pulmonary Langerhans' cell histiocytosis, diffuse pulmonary Rosai Dorfman disease [61], pulmonary lymphangitic carcinomatosis [62], pulmonary malakoplakia and Hermansky-Pudlak syndrome [34]. The detection of CD68(+), CD1a(−) histiocytes in bronchoalveolar lavage fluid confirms the diagnosis of pulmonary ECD [26]. Common symptoms of pulmonary ECD include a dry cough and insidious dyspnea which progresses over a period of months to years [61]. Cyanosis is less frequently described. Pulmonary function tests in patients with ECD typically show a mild restrictive ventilation pattern with normal or reduced carbon monoxide diffusion capacity [34]. Arterial blood gas values are usually normal [26]. Nevertheless, hypoxia and hyper- or hypocapnia may occur due to disease progression [34]. Arnaud and colleagues researched the pulmonary manifestations of ECD in their retrospective analysis of 34 ECD patients. They reported that high resolution CT findings associated with ECD involve the lung's parenchyma and/or pleura. These findings include interlobular septal thickening, centrilobular micronodular opacities, thickening of the interlobar fissures, parenchyma consolidations, microcystic lesions, thin wall cysts, pleural effusion and pleural thickening. The histiocytic infiltration in cases of pulmonary ECD seem to follow a lymphangitic distribution pattern, affecting the visceral pleura, interlobular septa and around the bronchoalveolar bundles. This pattern is usually accompanied by fibrosis, hypothesized to be associated with the activity of factor XIIIa positive dendritic cells. Most interestingly, these cells are abundant in the connective tissue relating to sites of the lymphangitic distribution discussed above [61]. Several studies reported that pulmonary lesions in ECD exhibit immunopositivity for factor XIIIa [11,61]. Extensive infiltration and fibrosis of the lungs may instigate severe cardiopulmonary symptoms, consequently, leading to cardiorespiratory failure [61]. However, in the absence of extensive pulmonary involvement, these symptoms may arise secondary to cardiac involvement promoting cardiogenic pulmonary edema [26]. Inspection of the caliber of the pulmonary veins may reveal whether the cause of cardiopulmonary compromise is of pulmonary or cardiac origin: a normal caliber would likely indicate a pulmonary cause and an extended caliber - a cardiac cause. Despite all of the above, in a study performed by Arnaud et al., the researchers concluded that pulmonary involvement of ECD has a limited impact on the overall prognosis of the disease [26].

### Retroperitoneal and renal involvement

The retroperitoneal space is one of the target destinations of histiocytic infiltration in ECD. We already discussed the perivascular infiltration affecting the abdominal aorta. The appearance of a "coated aorta" on CT may often appear in conjunction with infiltration and fibrosis of lower retroperitoneal structures, namely the adrenal glands, kidneys, renal arteries, ureters and adjacent anatomical spaces and borders [52]. Overall, ECD associated involvement of the retroperitoneal space was reported in 68% of the patients [1]. The majority of cases in which retroperitoneal involvement appears are asymptomatic [63]. The differential diagnosis of ECD associated retroperitoneal fibrosis includes idiopathic retroperitoneal fibrosis (also known as Ormonds' disease) and secondary retroperitoneal fibrosis [25,64]. When present, the symptoms are dysuria and abdominal pain. Large palpable kidneys may be detectable on physical examination [63]. Infiltration of the perirenal fat appears as an irregular renal border producing a "hairy kidney" appearance on CT. This finding undergoes enhancement after the administration of iodinated contrast material and can thus be differentiated from the kidney itself [65]. A massive infiltration of the perirenal space was reported by Wimpissinger et al., presumably compressing the kidneys and causing progressive renal failure. This condition was successfully alleviated by an open surgical approach, aimed at reducing the amount of tissue encapsulating the kidney and relieving pressure [66]. Perirenal infiltration may progress to the renal sinuses and produce a post renal obstruction. The infiltrates and subsequent fibrosis may also cause bilateral ureteric obstruction resulting in hydronephrosis and compromise of renal function. The ureteral segments which are most commonly affected by the fibrosis are the middle and distal segments [25]. As previously mentioned, the renal arteries may also be subjected to infiltration and fibrosis. Stenosis of the renal arteries, causing decreased renal perfusion will consequently instigate a state of renovascular hypertension via a renin-angiotensin mediated pathway. CT in conjunction with angiography proves as a valuable tool in the assessment of ECD related perivascular fibrosis [63]. Infiltration of the adrenal glands and fossae appears in the vast majority of patients with a multisystemic disease. It is predominantly bilateral in nature, although unilateral adrenal infiltration has been reported. In a case series of 7 patients reviewed by Haroche and colleagues one patient was found to experience adrenal insufficiency due to ECD related adrenal involvement [67].

### Exceptional sites of involvement

Involvement of the skin [4,5,68], gastrointestinal tract [69-71], testes [72], thyroid [72], skeletal muscle [73] and breast [74,75] were anecdotally reported. Involvement of the skin seems to be the most common among the rare presentations of ECD. In the retrospective study performed by Veyssier-Belot, 11 out of 59 patients presented with ECD associated cutaneous involvement [5]. Among the most common dermatological presentations of ECD are xanthoma like papules [68] and periorbital xanthelasma like skin lesions [4,44,61].

### Laboratory findings in ECD

Laboratory findings in ECD are non specific, non diagnostic and may serve to complement the backbone of radiological and histological findings. These are usually intertwined with the general symptoms of the disease and when apparent, they also may serve as means of evaluating the patient's well being and provide gross assessment of the patient's response to treatment. Laboratory findings of various ECD patients may include elevated erythrocyte sedimentation rates (ESR), increased levels of alkaline phosphatase and increased levels of C-reactive protein (CRP) [5,9,28]. More specific laboratory findings are used for the assessment of various physiological functions affected by multisystemic ECD and could better direct towards comprehensively understanding the patient's distribution of the disease. Within the settings of ECD, elevated serum creatinine and uric acid are suggestive of renal involvement. Increased levels of prolactin alongside decreased levels of LH, FSH, ACTH, GH, TSH and alteration of other hypothalamic-pituitary axis associated hormones are suggestive of ECD related pituitary insufficiency. Elevated serum osmolality in conjunction with appropriate water deprivation test results are suggestive of ECD associated diabetes insipidus [13].

### Imaging studies and radiology: initial diagnosis vs. follow-up

The initial step towards a diagnosis of ECD is either ^99m^Tc bone scintigraphy [76] and\or radiography findings that are virtually pathognomonic to the disease. Conventional radiography usually demonstrates cortical thickening with a reduced corticomedullary cavity and scintigraphy exhibits increased tracer uptake: both highlighting the symmetric bilateral diametaphyseal osteosclerosis [30]. Yet, considering that most patients have multiple (skeletal and extraskeletal) sites of involvement at presentation [4] follow-up imaging presents with a more complex problem: performing a panoramic assessment of the progression of multifocal lesions. None of the common imaging modalities that are used in ECD (i.e., radiography, ^99m^Tc bone scintigraphy, computed tomography (CT) and magnetic resonance imaging (MRI)) is able to provide a global assessment of the lesions during a single session [77]. Arnaud et al. researched the application of PET scans in ECD and concluded that PET scan assessments of osseous involvement reveals typical bilateral and symmetric uptake of FDG in the long bones similar to that observed with ^99m^Tc bone scintigraphy. Furthermore, they concluded that a whole-body PET scan is able to depict simultaneously many of the most relevant lesions encountered among ECD patients. After thoroughly reviewing 31 cases of ECD in their retrospective study, Arnaud et al. reported that PET scan sensitivity varies greatly among the different sites of involvement studied, but shows excellent specificity when compared with most other imaging modalities. Thus, suggesting that PET-CT is the best modality during follow-up of the disease [77]. PET-CT also serves as a valuable tool in the initial steps of diagnosis: it may assist in assessing the bone marrow involvement of the patient and identify candidate areas for CT guided percutaneous biopsy when extraskeletal involvement seems prominent [28]. High risk infiltration sites should be routinely monitored in ECD. MRI is the modality of choice when evaluating the different CNS manifestations. MRI should be used for the overall assessment of cerebral and cerebellar lesions, the hypothalamic pituitary axis and the orbits [10]. The efficacy of whole body MRI has not yet been fully evaluated, yet preliminary results seem promising [78]. CT complements the MRI findings by adding data concerning the involvement of the skull bones and sinuses. After researching the aspects of cranial imaging in ECD patients, Drier et al. recommend to systematically perform cerebral MRI on ECD patients. This is due to the severity of potential progression and risks associated with perivascular lesions and intracranial vascular stenosis [10]. Cardiac and mediastinal infiltrations are assessed primarily using contrast enhanced CT. Moreover, cardiac gated multi-detector CT can better demonstrate the infiltrate sheathing of the aortic root and proximal portions of the coronary arteries [65]. This can also be seen on cardiac MRI [54]. Echogradiography can add valuable data concerning the extent of cardiac involvement and of pericardial effusion in particular [49]. Both CT and MRI are useful tools for the assessment of the thoracoabdominal involvements of ECD. Retroperitoneal involvement, periaortic ("coated aorta") and perirenal ("hairy kidney") in particular can be observed on both enhanced and unenhanced CT scans. These can be complemented by angiography studies for the evaluation of possible stenosis due to perivascular infiltration, a possible complication of renal artery involvement. A multi-detector CT scan can be used for better representation of the renal pelvises, enabling detection of distal ureteral obstructions and hydronephrosis [65]. Renal duplex ultrasound is another option for an assessment of the renal arteries and ureters and may also assist in gross kidney measurements [63]. Retrograde pyelography can be used preoperatively to assess the compression on the urinary tract [66]. Pulmonary assessment includes high resolution CT for the detection of lung or pleural involvement. Arnaud et al. suggest using high resolution CT at the time of diagnosis, during initial assessment and later on if pulmonary symptoms occur. If necessary, this should be done in conjunction with an annual assessment using a low dose CT of the chest, abdomen and pelvis, closely examining the lung window for ECD associated pulmonary findings [26]. Rarer breast involvement can be seen on mammography [75].

### Pathology

In order to finalize a diagnosis of ECD, a histopathological confirmation is necessary. A biopsy is usually obtained from bone, skin, retro-orbital or retroperitoneal soft tissue [13]. Confirmation is made upon detection of CD68(+), CD1a(−) non Langerhans histiocytes with foamy or eosinophillic cytoplasm lacking Birbeck granules. These are usually accompanied by a microscopic environment including polymorphic granulomae, fibrosis, xanthogranulomatosis, proliferating fibroblasts, lymphocytic aggregates and Touton giant cells.

### Pathogenesis of ECD

The CD34(+) myeloid stem cell gives rise to three lineages of histiocytic and dendritic cells [27]. While Langerhans' cell histiocytosis exhibits the proliferation of CD1a(+), Langerin(+), S100(+) Langerhans' dendritic cells, ECD is hypothesized to originate from the proliferation and migration of CD68(+), CD1a(−) non Langerhans' histiocytes of monocyte-macrophage descent. Discussions concerning the interplay between Langerhans' cell histiocytosis and ECD in face of patients with both diseases yielded no conclusive answers. Some researchers hypothesized that the existence of patients with both diseases is attributed to an unknown abnormality or dysfunction of the CD34(+) progenitor cell. ECD differs from Langerhans' cell histiocytosis by the immunohistologic and microscopic characteristics of the histiocytes involved. Langerhans-like histiocytes stain positive for S-100 protein, and electron microscopy of their cytoplasm discloses Birbeck granules in more than 20% of cells [79]. ECD histiocytes have neither of these characteristics [5]. However, cases in which S-100 expression was positive were observed in the literature [61]. The typical histological appearance of ECD consists of CD68(+), CD1a(−), S-100(−/low) non langerhans cell histiocytes. Moreover, Factor XIIIa was found to be positive in ECD lesions derived from pulmonary origin [61]. The microscopic environment of the lesion includes xanthogranulomatosis, proliferating fibroblasts (generating fibrosis), lack of eosinophils, lymphocytic aggregates and Touton giant cells [13,26,37]. Recent studies attempted to elucidate the pathogenesis of ECD. It is currently unknown whether ECD is a monoclonal neoplastic process or a polyclonal reactive process. Several researchers attempted to provide a solid answer to that question, some utilizing human androgen receptor gene assays (HUMARA) and some searching for cytogenetic clonal abnormalities [21,80-82]. Alas, due to conflicting data, no definite answer was found. A different approach proved more fruitful. A recent study of 37 patients by Arnaud et al. identified a cytokine\chemokine profile unique to ECD, consisting of increased levels of interferon-α, interleukin-12, monocyte chemotactic protein-1 (MCP1/CCL2) and decreased levels of interleukins 4 and 7. This profile is constant despite treatment with interferon-α, suggesting that interferon-α has a limited role in altering the cytokine network at hand. Most interestingly, both interferon-α treated and untreated patients exhibited high levels of interferon-α. Moreover, the paradoxical high levels of interferon-α seem to originate from an unknown source, as no interferon-α secreting dendritic cells were identified in ECD lesions. However, the lesions stained positive for interferon-γ. Intralesional interferon-γ positive lymphocytes and the detection of interferon-γ inducible protein 10 (OP-10) expressed by histiocytes provided evidence that ECD is characterized by a strong Th1 immune response [83]. Stoppacciaro et al. also reviewed chemokine and cytokine levels in three ECD patients. They reported an increased expression of chemokines and their corresponding receptors in ECD histiocytes and in epithelial cells of vessels inside the lesions. Also, they reported that Ki-67 staining was undetectable and that no mitotic figures were seen adding evidence against a mechanism of intra-lesional histiocytic proliferation. Evidence of a Th1 immune response also appears in this study as high levels of interferon-γ with low levels of interleukin-10 were reported [84]. Several studies indicated increased levels of TNFα [85], interleukin-6 [86] and normal to high levels of interleukin-1β [9,83,87,88]. Future studies are needed to assess the importance of these cytokines as therapeutic targets. The anecdotal cases which describe patients who were treated with infliximab and anakinra based on these therapeutic targets showed optimistic results. Finally, recent data points to a high prevalence of BRAF V600E mutations among ECD and LCH patients [18,89] advocating the possible usage of BRAF inhibitors such as vemurafenib in the targeted treatment of ECD.

### Treatment

To this date, various treatments have been administered to ECD patients in an attempt to achieve remission or at least stabilization. Several approaches based on diverse biological theories and small scale clinical experience have been proposed. Currently, interferon-α provides the best management strategy, with sustainable stabilization of the disease in most cases, [1,12,90,91]. Interferon-α is administered at dosages ranging from 3 million units three times per week to 9 million units 3 times per week. Peginterferon alfa-2a, the pegylated form of interferon-α, is an equivalent alternative to interferon-α. It is administered at dosages ranging from 135–200 μg per week [1]. It must be taken into consideration that treatment with these agents is prolonged. Thus, tolerance to treatment should be considered pivotal in long term treatment planning. Among the adverse effects of interferon-α are asthenia, myalgia, pruritus, thrombocytopenia and depression [92]. The efficacy of interferon-α varies in concordance with the different sites of disease involvement. One recent large survey encompassing 24 patients treated with high dose interferon-α (>18 million units per week) or high dose peginterferon alfa-2a (>185 μg per week) reported them to be efficacious in the treatment of patients with severe ECD. The response to treatment was most prominent in the cutaneus foci of the disease, followed by involvement of the CNS, pituitary, lungs and heart, which comprise foci that are more resistant to treatment [92]. Several smaller reports corroborate the efficacy of interferon-α in respect to other sites of the disease. Braiteh et al. report substantial durable regression (3–4.5 years) of retro-orbital lesions, as well as marked improvement in bone lesions, pain and symptoms of diabetes insipidus in three patients treated with interferon-α [90]. Arnaud et al. report interferon-α had only a minor impact on the course of pulmonary ECD involvement [26]. No interferon-α response predicting factors were identified [92]. However, in a recent survey of 53 patients, treatment with interferon-α itself was identified as a independent predictor of survival. It is currently the only agent that has shown to improve survival among ECD patients [1]. Cladribine (2cda) is advocated as an alternative treatment for ECD. However, the literature contains significantly less data regarding this drug in comparison to interferon-α. Cladribine is administered at dosages ranging from 0.07-0.14 mg/kg/day for five consecutive days. Myra et al. reported an ECD patient who experienced marked recovery pursuant treatment with cladribine [93]. Adam et al. reported partial regression of CNS ECD lesions following treatment with a cladribine based regimen [94]. As for adverse effects, cladribine may be associated with dose dependent bone marrow suppression, and neurological toxicity. Sheidow et al. reported a single ECD patient who developed sudden onset bilateral blindness presumably due to cladribine related toxic injury to the optic nerves. Corticosteroids carried the promise of immune suppression, but proved to have a very limited impact on the disease [1,5,90]. Different types of chemotherapy based regimens were attempted with various degrees of success, but in most cases provided only temporary relief [5,44,93,95]. Radiation therapy fails to yield a sustainable clinical response [90]. Surgical debulking may provide a temporary solution for specific compromising scenarios, as the lesions tend to re-grow rapidly [13]. Inhibition of PDGF signaling using imatinib or sunitinib yielded modest results at best [26,48,96]. Treatment with bisphosphonates produces only partial success in the management of osseous involvement [86,97,98]. Recent advancements in the understanding of the molecular biology of ECD presented with new promising treatment options. Encouraging results accumulate regarding the targeting of interleukin-1 (Anakinra) [9,88,99,100]. One other promising pharmaceutical agent is the BRAF inhibitor vemurafenib, which recently exhibited dramatic efficacy in the treatment of three ECD patients whose histiocytes were positive for the V600E BRAF mutation [101]. Finally, other possibilities based on the immunological basis of ECD include anti interleukin-6 therapy (Tocilizumab) and anti TNFα therapy (Infliximab) [85,102].

### Summary and future perspectives

Herein we have gathered a fair share of the scientific knowledge regarding ECD. A synopsis of the core data can be found in Table 2. As an orphan multi-systemic disease both diagnosis and treatment are challenging. Diagnosis wise, the challenge is lesser and requires a high degree of suspicion. Treatment and management of the disease are of greater complexity. Since no definite cure exists, the goals of treatment should be prolonging life and maximizing their quality. Psychological consulting is important because success of the physical treatment usually results in the maintenance of a chronic condition. As such, it may be accompanied by various difficulties, deficits and secondary complications. The physical component of treatment should be supervised by a multidisciplinary team of specialists, whose expertise should correlate with the patient's distribution of the disease. Secondary intervention and treatment modifications should be made based on proper assessment of the disease's progression as well as the patient’s well being. With time, better understanding of the immunology and molecular biology that underlie this condition will ultimately lead to the emergence of novel therapeutic approaches. Already, much progress had been made in identifying disease related elements which may prove pivotal for future management. One such example is the identification of the cytokine network patterns that characterizes ECD and consequently, the possibility of treatment via a mechanism of pro-inflammatory cytokine inhibition. Another example is the identification of mutated cellular components which may be responsible for the proliferation and differentiation of ECD histiocytes. Possibly, such is the case of the V600E BRAF mutation and vemurafenib.

**Table 2:** Profile of the ECD patient

Age at onset	5th - 7th decade of life	Arnaud et al. [1]
Presenting symptoms	Diabetes insipidus, bone pain and exophthalmos	Veyssier-Belot [5]
Disease distribution	**Skeleton:** Long bones osteosclerosis (bone pain)	Dion et al. [30]
	**Cranium** &**CNS:**	Drier et al. [10]
	Pituitary defects (diabetes insipidus, panhypopituitirism)	
	Retro-orbital lesions (exophthalmos)	
	Cerebellar lesions (ataxia)	
	**Cardiovascular:**	Haroche et al. [54]
	Pericardial infiltration / effusion	
	Periaortic sheathing ("coated aorta")	
	Myocardial infiltration / right atrial tumor	
	**Pulmonary:**	Arnaud et al. [26]
	Interstitial lung disease	
	Pleural effusion	
	**Renal** &**Retroperitoneal:**	Haroche et al. [2]
	Perirenal infiltration ("hairy kidney")	
	Post renal obstruction (hydronephrosis)	
	Renal artery stenosis (renovascular hypertension)	
	**Cutaneous:** Periorbital xanthelasmae	Veyssier-Belot [5]
Imaging	^99m^Tc Bone Scintigraphy	Gotthardt et al. [76]
	PET/CT	Arnaud et al. [77]
Treatment	**1st line alternatives:**	Arnaud et al. [1]
	Interferon-α / Peginterferon alfa-2a	Hervier et al. [92]
	**2nd line alternatives:**	
	Cladribine^*^	Myra et al. [93], Adam et al. [103]
	Vemurafenib	Haroche et al. [101]
	Anakinra	Aouba et al. [88]
	Infliximab	Dagna et al. [85]
Prognosis	**1 year survival:** 96%	Arnaud et al. [1]
	**5 year survival:** 68%	

(*) may be advocated as a first line treatment modality among some specialists.

## Abbreviations

ECD: Erdheim chester disease; LCH: Langerhans cell histiocytosis; JXA: Juvenile xanthogranuloma; 99mTc: Metastable nuclear isomer of technetium-99; CNS: Central nervous system; MRI: Magnetic resonance imaging; ECG: Electrocardiography; CT: Computed tomography; CRP: C-reactive protein; ESR: Erythrocyte sedimentation rate; LH: Luteinizing hormone; FSH: Follicle-stimulating hormone; ACTH: Adrenocorticotropic hormone; GH: Growth hormone; TSH: Thyroid stimulating hormone; PET: Positron emission tomography; FDG: Fludeoxyglucose; HUMARA: Human androgen-receptor gene assays; MCP1: Monocyte chemotactic protein-1; OP-10: Interferon-γ inducible protein 10; TNFα: Tumor necrosis factor alpha; 2CDA: Cladribine.

## Competing interests

The authors declare that they have no competing interests.

## Authors’ contributions

RDM, MMM & YS participated in the process of the literature review and in the drafting the final manuscript. In addition, YS supervised the project. All authors read and approved the final manuscript.
